# Exploring the dynamic functions of pastoral traditional knowledge

**DOI:** 10.1007/s13280-025-02131-x

**Published:** 2025-02-01

**Authors:** Ouerle Chao, Xiaoyue Li, Victoria Reyes-García

**Affiliations:** 1 Institute of Environmental Science and Technology, Universitat Autònoma de Barcelona (ICTA-UAB), 08193 Bellaterra, Barcelona, Spain; 2 Institució Catalana de Recerca i Estudis Avançats (ICREA), 08010 Barcelona, Spain; 3 Department d’Antropologia Social i Cultural, Universitat Autònoma de Barcelona, 08193 Bellaterra, Barcelona, Spain

**Keywords:** Knowledge functions, Pastoral traditional knowledge, Pastoralism, Resilience, Traditional ecological knowledge

## Abstract

Pastoral traditional knowledge is increasingly recognized for its inherent adaptability in addressing contemporary challenges and increasing the resilience of pastoral communities. To deepen our understanding of how this knowledge system demonstrates adaptive characteristics, we employ a functional lens to examine its dynamic nature in this systematic review. Our analysis, based on insights from 152 case studies worldwide, shows that this knowledge system has various domains and serves diverse functions, including ecological, economic, and socio-cultural functions, with further subfunctions. Ecologically, pastoral traditional knowledge predominantly enhances climate adaptation and resilience; economically, it helps maintain herd productivity and sustain livelihood support; and socio-culturally, it is crucial for preserving the cultural identity and heritage of pastoral communities. Furthermore, our findings highlight that each knowledge domain shows multifunctional characteristic. Our analysis also helps identify common functions across eight knowledge domains, each contributing to areas like sustainable resource management and climate adaptation, though to varying degrees.

**Supplementary Information:**

The online version contains supplementary material available at 10.1007/s13280-025-02131-x.

## Introduction

Rangelands, covering around 25–45% of the earth’s land surface, are remarkably diverse, including semi-arid savannas, grasslands, mountainous regions, and arid deserts (Reid et al. 2014). Within these varied landscapes, pastoral communities have developed rich and distinctive bodies of knowledge shaped by intimate and enduring interactions with the local environment (Reid et al. 2014; Sharifian et al. 2023; Tugjamba et al. 2023). These knowledge systems are rooted in traditions that have accumulated over generations and continue to evolve (Reyes-García et al. 2014). The dynamic nature of pastoral traditional knowledge (PTK) plays a crucial role in the survival of pastoral communities, enabling them to navigate various challenges and enhancing their resilience, understood as their capacity to absorb disturbance, maintain functionality, and adapt in response to change (Folke 2006; Arjjumend 2018; Ahmed et al. 2023).

Currently, pastoral communities face numerous challenges. Climatic variability has a considerable impact on pastoralists’ traditional ways of living and the natural environment they depend on. With advancing climate change, pastoralists are witnessing greater unpredictability in climatic patterns, more intense droughts, increasing land degradation, and declining pasture quality (Ifejika Speranza 2010; Ahmed et al. 2023). Socio-economic pressures such as market influence, urbanization, and extraction of natural resources have been exasperating the already overloaded adaptive capacity of pastoral communities (Galvin 2009). Beyond this, policies or conflicts regarding land ownerships also put further restrictions on traditional grazing for access to fundamental resources, aggravating these vulnerabilities (Xie and Li 2012). These ecological and socio-economic challenges threaten not only the ecological balance but also directly influence the livelihood and cultural continuity of pastoral societies.

Despite these challenges, pastoral communities persistently employ and renew their traditional knowledge, continually learning from and adapting to changing environmental conditions and socio-economic landscapes. In addressing the challenges posed by climate variability, pastoral communities have developed a diverse range of adaptive strategies that reflect the depth of their traditional knowledge. These include predicting weather patterns by observing animal behavior, plants, and natural phenomena, as well as employing practices such as herd mobility, herd diversification, and herd sharing (Arjjumend 2018; Radeny et al. 2019; Ochieng’ et al. 2020). For example, shamans of the Tukano people of Colombia monitor species abundance by randomly scheduling hunting excursions. Monitoring ecosystem changes is another key function of many traditional knowledge practices that can be seen among the Sahel herders who observe grazing pressure and the condition of pastures to inform their decisions on when to rotate or move their herds (Berkes et al. 2000). The practice of herd mobility also exemplifies how pastoralists manage rangeland ecosystems in a way that is sustainable. This key practice involves strategic movements of herds to alternate grazing areas, the movement being crucial for allowing time for degraded pastures to recover (Dominguez et al. 2010; Stammler-Gossmann 2010).

Besides its ecological significance, PTK also helps communities achieve significant economic benefits. For example, pastoralists have a rich understanding of the nutritional values of different plants. This includes knowing which plants induce milk production, which plants are the best for weight gain, and which others improve general livestock health (Sharifian et al. 2023). Using this specialized knowledge of forage/plant, pastoralists make informed decisions about when and where to graze their livestock (Fernández-Giménez 2000; Molnár 2017). Moreover, the geographical dispersion of pastoralist settlements across extensive grasslands, often isolated from one another, necessitates a reliance on the social–cultural dimensions of PTK. For instance, kinship-based social networks are instrumental in enabling pastoralists to exchange experiences, share resources, and mitigate risks (Salpeteur et al. 2015; Chao et al. 2023). Diversifying livestock species and breeds also functions as a risk management strategy, allowing pastoralists to manage various climate conditions as certain species or breeds show better drought tolerance, whereas others are more adapted to the cold (Ghorbani et al. 2013).

Beyond ecological and economic functions, PTK also plays essential roles in the preservation of the socio-cultural fabric of pastoral communities. The traditional knowledge, practices, and culture developed by pastoral communities in the management of their environments and existence primarily provide additional support to the socio-cultural continuity in the pastoral communities (Oteros-Rozas et al. 2013; Stolton et al. 2019). For example, the traditional customary laws and governance systems that regulate access to communal resources, such as pastures, can facilitate conflict resolution among different groups or families (Sundstrom et al. 2012; Kaoga et al. 2021). Moreover, herd breeding also brings forth these socio-cultural functions, as the selection of breeding stock is sometimes ritualistic and associated with traditional ceremonies and festivals, which enhance community ties and preserve heritage (Gandini and Villa 2003; Ahozonlin and Dossa 2020).

From climate-related knowledge to social–cultural wisdom, the application of the various components of PTK to tackle different challenges suggests the diverse functions that the knowledge system can play in pastoral life. While taken as a whole, the PTK system plays various functions at different levels of pastoral life, each knowledge domain within the system also shows multifunctional characteristic. For instance, the practice of herd mobility is not only instrumental in sustaining herd health, but it also serves as a crucial strategy for pasture preservation (Wario et al. 2016). Similarly, cultural practices are also multifaceted, playing a vital role not just in the maintenance of cultural identity and community cohesion but also in prompting ecological sustainability (Dominguez et al. 2010; Ghorbani et al. 2013; Chao et al. 2023). Similarly, traditional forage/plant-related knowledge not only contributes to livestock productivity and, therefore, determines economic stability but also supports ecological management through fostering sustainable grazing practices (Sharifian et al. 2023). Moreover, this knowledge sometimes holds cultural importance, appearing in traditional healing practices and rituals (Frascaroli et al. 2014).

In addition to the multifunctional characteristic within each domain of PTK, different knowledge domains synergistically contribute to achieving common objectives and outcomes. Traditional knowledge systems are holistic and complex (Berkes et al. 2004; Berkes 2011). Different types of knowledge do not exist and operate in an isolated way; rather, they are interconnected. For instance, if unexpected weather changes occur, communities rely on not solely their knowledge about climate and weather (Radeny et al. 2019); they also utilize their knowledge about landscapes (Wangdi and Norbu 2018), forage/plant (Fernández-Giménez et al. 2021), livestock (Shukurat et al. 2012), and even spiritual rituals (Roncoli et al. 2001) to adapt to the changes from different angles holistically.

To dig deeper into how PTK helps pastoralists adapt and thrive amid changing environmental and socio-economic pressures, we decided to apply a functional lens in this study. The function of knowledge, within this context, refers to the deliberate use of knowledge to achieve specific objectives as well as the actual impacts or outcomes of the knowledge. Specifically, we review the literature (1) to document the domains of PTK, (2) to explore the diversity of functions PTK serves in the lives of pastoralists, (3) to investigate whether a single PTK domain can serve multiple functions, and (4) to examine whether different PTK domains share common functions. The current literature acknowledges the contribution of the multifunctional nature of PTK in climate adaptation (Reid et al. 2014) and sustainable management of rangeland and livestock (Oba 2012), but to date, no study has explicitly and systematically reviewed PTK through a functional lens.

## Methodology

### Selection of publications

Our review used primary sources from peer-reviewed literature on PTK from the Web of Science and Scopus. The search was conducted in July 2022 and included publications up to that date. To ensure a comprehensive exploration of PTK and its functions, we developed a detailed search string that encompassed terms that explain the dynamic functions of PTK. This choice helped us to explore how PTK serves multiple purposes in different contexts, especially under different environmental and socio-economic pressures. The search string used was TS = ((traditional OR Indigenous OR local OR past OR old OR folk OR aborigin) AND (pastoral* OR nomad* OR herd* OR shepherd OR flock) AND (ecology* OR environment* OR rangeland OR grassland) AND (knowledge* OR practice* OR strategy*)) AND TS = (adapt* OR cop*).

The initial search yielded 1076 documents (WoS = 432; Scopus = 644). After removing overlapping documents based on DOI, title, and abstract comparisons (n = 318), we thoroughly screened 758 publications for relevance to our review, focusing on title, abstract, and methods. During the screening process, we followed strict criteria to ensure the quality and relevance of selected studies. We included only peer-reviewed studies that provided empirical evidence of PTK and had a clear focus on PTK. Studies that only partially addressed PTK were included if the relevant section to PTK provided detailed insights. Publications were restricted to those available in English or Chinese. The inclusion of Chinese-language studies is tied to the first author’s ability to speak Chinese. This linguistic focus, however, introduces a potential bias by excluding significant works published in other languages, especially from other regions where pastoralism is prevalent, such as Africa and Central Asia. While the concept of adaptation was a significant aspect of our search, its explicit mention was not a prerequisite for inclusion. This approach allowed us to consider a broader spectrum of documents that offered valuable insights into the diverse functions of PTK, extending beyond mere adaptive responses. Ultimately, 149 papers met our criteria and were included in the review. The list of publications included is presented in the supplementary material (Table S1).

### Data collection

During the data collection phase, each document was assigned a unique ID. For those documents containing multiple case studies, data for each case were collected separately. We recorded the geographical locations of each case study and climate zone using the Köppen-Geiger climate classification system. We also documented the characteristics of the study populations. This included noting their ethnic names, as mentioned in the original articles, and assessing whether they were identified as indigenous groups by the authors of the publication.

Additionally, we documented the types of livestock managed and the various pastoral practices of these communities. We distinguish among *nomadism*, characterized by the regular movement of herds to new pastures; *transhumance*, involving seasonal migration between fixed pastures; *agro-pastoralism*, which combines crop farming with pastoralism; and *sedentarism*, indicating settled herding with limited livestock movement. Verbatim statements referring to PTK and its application were recorded from each selected paper. While all selected statements were directly relevant to the following aspects: the application of PTK, the motivations for its application, and the outcomes of associated knowledge practices, some relevant quotes might be unintentionally excluded.

### Data analysis

To document PTK domains, we conducted qualitative thematic analysis and coded the verbatim statements referring to PTK in Nvivo (Table S2). We categorized the extracted data into several knowledge domains based on a classification framework from Sharifian et al. (2022), which organizes PTK into the following domains: livestock, forage/plant, landscape, climate/weather, and social–cultural knowledge. During the coding process, we created additional cross-knowledge practices based on frequency of occurrence. For example, in our study, we distinguish ‘herd mobility practice’ and ‘herd diversification practice’ from the broader ‘livestock-related knowledge’ domain. This distinction is based on our understanding that these domains not only involve knowledge of the livestock but also encompass a comprehensive understanding of landscape, forage/plant, and climate/weather.

To investigate the diverse functions of PTK within pastoralist communities, we coded quotes from the selected cases using the qualitative data analysis software Nvivo (Table S2). Each quote was coded based on its thematic content and relevance to specific functional categories. Initially, all quotes relevant to PTK and its applications were classified into three main categories used in social–ecological systems research (Folke et al. 2002; Boström 2012): ecological function, economic function, and social–cultural function.

Within the three functional categorizations, we identified 10 subfunctions of PTK using an inductive qualitative content analysis. To develop these subcategories, we thoroughly reviewed the verbatim statements of PTK functions extracted from the publications and applied Nvivo coding to identify emerging themes.

Furthermore, to ensure accuracy and reliability, following the initial coding process by the lead author, the two other co-authors independently reviewed the codes assigned by the primary coder. To address coding discrepancies identified during the independent reviews, including whether to separate ‘herd mobility practice’ and ‘herd diversification practice’ from the livestock-related knowledge domain, we had a meeting where we examined each issue raised by the second and third authors. In cases where a consensus could not be reached, the first author made the final decision.

Additionally, we quantitatively assessed the distribution of the coded functions among different domains using the R program, specifically ‘dplyr’ for descriptive analysis and ‘ggplot2’ for data visualization. Specifically, to assess the diverse functions of PTK, we first counted the mentions of knowledge that contributes to ecological, economic, and social–cultural functions. Then, for each main functional group, we calculated the percentage of different knowledge domains contributing to that group. Furthermore, we conducted a detailed analysis to understand the weight of different subfunctions within the three main functions. We categorized the knowledge that plays ecological functions into sub-ecological groups. The same approach was applied to the economic and social–cultural function categories.

Using ‘dplyr,’ we evaluated the distribution of different functions across various PTK domains and analyzed how these knowledge domains contribute to various functions by calculating the percentage distribution within each sub-functional group. The common functions among these domains were also assessed. This involves calculating the frequency and percentages with which different domains (livestock, forage/plant, landscape, climate/weather, social–cultural, mobility, herd diversification, and other) contributed to the same function. Visualization with ‘ggplot2’ offered insightful representations, including pie charts and bar charts to depict PTK functions and stacked bar charts to illustrate the distribution of functions within each domain.

Additionally, to comprehensively understand the distribution of case studies, practiced pastoral types, and pastoral knowledge domain across varying climate zones, spatial analysis was conducted using ArcGIS Pro.

## Results

### Overview of the case studies

**Fig. 1:**
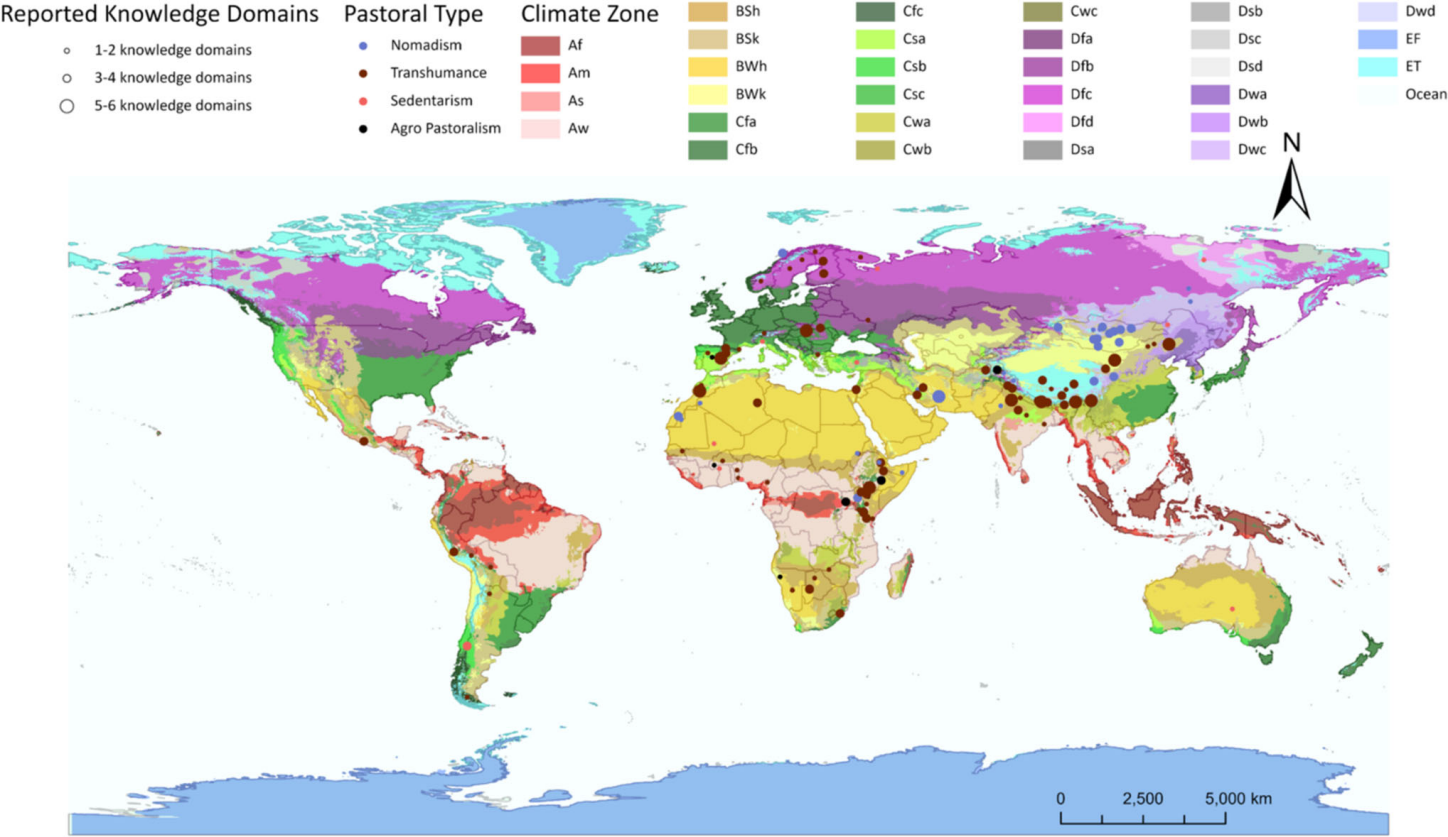
Global distribution of the case studies (n = 152), pastoralism types, and pastoral knowledge domains integrated with the Köppen-Geiger climate classification

Our review includes 149 papers and 152 case studies from 62 countries, spanning all five main climate zones (Fig. 1). Case studies documented in our review are predominant in the dry climate, representing 38% (58 cases) of the total case studies. Following closely, cases in the temperate climate account for 24% (37 cases). The continental (22 cases) as the tropical climate (23 cases) also represents a considerable portion of the dataset, with approximately 15% of the cases in each climate zone. Lastly, 12 cases (8%) are found in the polar climate.

In terms of pastoral practices, transhumance is the most prevalent, comprising 66% (101 cases) of the cases, followed by nomadism with 28% (42 cases). Agro-pastoralism and sedentarism together account for 6% (9 cases). In the analysis of pastoralism types across climate zones, nomadic groups are predominantly found in dry climates, representing 44% (27 cases) within this climate zone. Transhumance, however, is the leading pastoral practice in a variety of climates, with majorities in the temperate (33 cases, 83%), continental (13 cases, 59%), tropical (18 cases, 64%), and polar climates (9 cases, 64%). In contrast, sedentary practices and the combination of transhumance and agro-pastoralism are less prevalent and do not dominate any specific climate zone.

Our compilation includes case studies from 81 different ethnic groups, of which 75 are identified as indigenous peoples in the original reference. While the dataset features a range of ethnic groups, some of the more prominent ones are Mongolian (17%), Tibetan (5%), Sámi (3%), Borana (3%), and Maasai (3%). In the studied cases, pastoral communities manage different animals: cattle are reported in 65% of cases, sheep in 73%, goats in 66%, camels in 29%, horses in 29%, reindeer in 9%, and yaks in 15%. Interestingly, 28 cases (18%) exclusively focus on single-species herds, predominantly cattle. In contrast, most communities (n = 124; 82%) employ mixed herding strategies. The mixed herding strategy is most prevalent in dry climates, where we find 55 cases of mixed herding and three cases of single-species herding. Similarly, the temperate and polar climates also tend to favor mixed herding strategies (37 vs. 30 cases for temperate and 12 vs. 9 in polar). In continental and tropical climates, there is a more balanced distribution between single-species and mixed-species herds.

### Domains of PTK

Results from our analysis expand upon the initial framework proposed by Sharifian et al. (2022), who identified five key knowledge domains: livestock, forage/plant, landscape, climate/weather, and social–cultural knowledge by adding two new domains to this classification: herd mobility practice and herd diversification practice (Table 1). The distribution of different knowledge domains across case studies shows a significant variation (Fig. 1). More than half of the case studies (84, 55%) reported only one or two knowledge domains, while 39% (59 cases) documented three to four domains. Notably, only 9 cases (6%) documented a range of five to six knowledge domains.

**Table 1:** Pastoral traditional knowledge domains

Knowledge domains	Definition	Frequency and percentage	Example quote and reference
(a)			
Livestock-related knowledge	This knowledge includes understanding the nutritional needs, well-being, grazing preferences, and unique characteristics of the livestock	40 cases; 26%	“They learn step by step how to identify their livestock from the rest, how to guard them and keep them healthy, how to herd them by using different signals and sounds.” (Malhotra et al. 2022, p 10)
Forage/plant-related knowledge	This knowledge involves the identification of plants, a detailed understanding of their characteristics, and their utilization	40 cases; 26%	“They also plant bechna (Panicum miliaceum L., millet), a cereal crop widely used as fodder and food. The Tuareg mostly appreciate millet’s nutritional value as fodder for sheep and goats.” (Miara et al. 2022, p 6)
Landscape-related knowledge	This knowledge refers to the traditional insights and techniques developed for interacting with and understanding specific landscapes	33 cases; 22%	“The Evenki topographic typology is a detailed collection of concepts that define mountains, hills, inclines, slopes, rivers basins, types of soil, snow specificities, etc. The socio-economic importance of topographic landscapes is huge to the nomads, as accessibility of these landscapes constitutes their richness.” (Lavrillier and Gabyshev 2021, p 1913)
Climate/weather-related knowledge	This knowledge refers to information about the local climate, weather patterns, and seasonal variations that impact pastoral activities	63 cases; 42%	“Some respondents indicated that they use different forecasting methods to predict the rain, such as bird movements, certain species of trees, wind patterns, phenology, presence and absence of certain animals, wind movements, moon and sun as indicators of whether they will have rain or not.” (Inman et al. 2020, p 11)
(b)			
Social–cultural knowledge	This knowledge is about the cultural traditions, social institutions, and community dynamics specific to pastoral societies	44 cases; 29%	“Adaptation strategies were mainly facilitated by local communal institutions, such as reciprocity and trust. The communal institutions were critical for climate adaptation in the remote Tibetan pastoral areas where local markets and economies were less developed.” (Wang et al. 2016, p 7)
Herd mobility practice	This practice refers to the movement of livestock groups from one location to another for different purposes	124 cases; 82%	“The second traditional management strategy is called “taking otor” in the Mongolian Language. During severe droughts, which affect large areas, herders would move temporarily over long distances to unaffected or less severely affected areas, perhaps even outside of the Alxa Left Banner. When local conditions improved, they returned to their original land. “(Zhang et al. 2013, p 184)
Herd diversification practice	This knowledge focuses on the trade-offs of diversifying the composition of the livestock	23 cases; 15%	“The herd’s composition and diversity makes it possible for the animals to graze on several species of plants at the same time, which decreases pressure on the palatable plants in one area and also prevents the extinction of unpalatable species.” (Ghorbani et al. 2013, p 12)
Other	This knowledge is about the cultural traditions, social institutions, and community dynamics specific to pastoral societies	17 cases; 11%	“By distributing livestock among a number of people in different places, pastoralists have been able to reduce the risks of livestock mortality during drought. This livestock loan system, called mafisa, is unique in southern Africa” (Reed et al. 2007, p 252)

Among the knowledge domains documented, herd mobility is a practice most frequently reported, identified in 82% of cases (124 cases) across all climate zones. For example, nomadic movement is widely practiced by herders in Mongolia (Tugjamba et al. 2021). Knowledge associated with other types of mobility, such as the rotational grazing practiced by the Sámi people in Finland, was also recorded (Turunen et al. 2016).

Climate and weather-related knowledge is the second highest cited knowledge domain, reported in 41% of the cases (n = 63). This knowledge is particularly crucial and widely applied in the dry climate zone (40%). Since many of the pastoralist communities reside in regions with highly variable climates, they have developed knowledge to predict the upcoming weather through observing natural phenomena, as well as changes in animal behavior and plants (Oteros-Rozas et al. 2013; Klein et al. 2014; Guoping et al. 2021). These observations include changes in rainfall, snow patterns, wind behavior, and growing seasons (Fernández-Giménez and Fillat 2012; Ghazali et al. 2022).

Social–cultural knowledge was highlighted in 29% of the cases (n = 44), with the presence of local institutions being the most frequently mentioned component. For example, in Nariyan, Iran, the indigenous institution known as *chakaneh* enables pastoralists to share responsibilities and resources, such as livestock management and access to grazing lands (Ghorbani et al. 2013). Similar local institutions are also found in some other pastoralist communities (Postigo 2020; Fernández-Giménez et al. 2021).

Many pastoral communities have deep connections with plant species in general and pastures in particular, which allows them to identify whether a pasture is good or bad, and the functions of different plants. Forage/plant-related knowledge was mentioned in 26% of the cases (n = 40), particularly in the temperate climate zone (38%). For example, pastoralists in Shilingol League, Mongolia, have an extensive understanding of plant characteristics relevant to herding, including their seasonal edibility for different livestock (Huang et al. 2009).

Similarly, pastoral communities hold rich livestock-related knowledge, or knowledge emphasizing the understanding of the specific needs and characteristics of livestock. This knowledge is reported in 40 cases (26%) and is particularly prominent in the dry climate zone (41%). As an example, the Borana pastoralists in Ethiopia have knowledge about the camel’s unique capabilities, including its resilience to water scarcity, feed shortages, heat stress, and drought conditions (Megersa et al. 2014). Tibetan pastoralists in Yunnan, China, are adept at recognizing signs of disease or nutritional deficiencies in their livestock through behavioral and physical cues (Haynes and Yang 2012).

Knowledge domains relatively less often mentioned in the literature include landscape-related knowledge and herd diversification practice. Landscape-related knowledge was found in 33 cases (22%). Pastoralists routinely observe and learn about their surroundings while herding. For instance, people living in Ansó, Spain, are cognizant of the changes happening in their landscape, such as the increase in shrub and forest cover (Fernández-Giménez 2015). Herd diversification practice appeared in 24 cases (15%). The Maasai pastoralists in Kenya, for instance, advocate for herd diversification, arguing that this strategy optimizes resource use and helps mitigate risks (Kaoga et al. 2021).

### Diverse functions of PTK

**Fig. 2:**
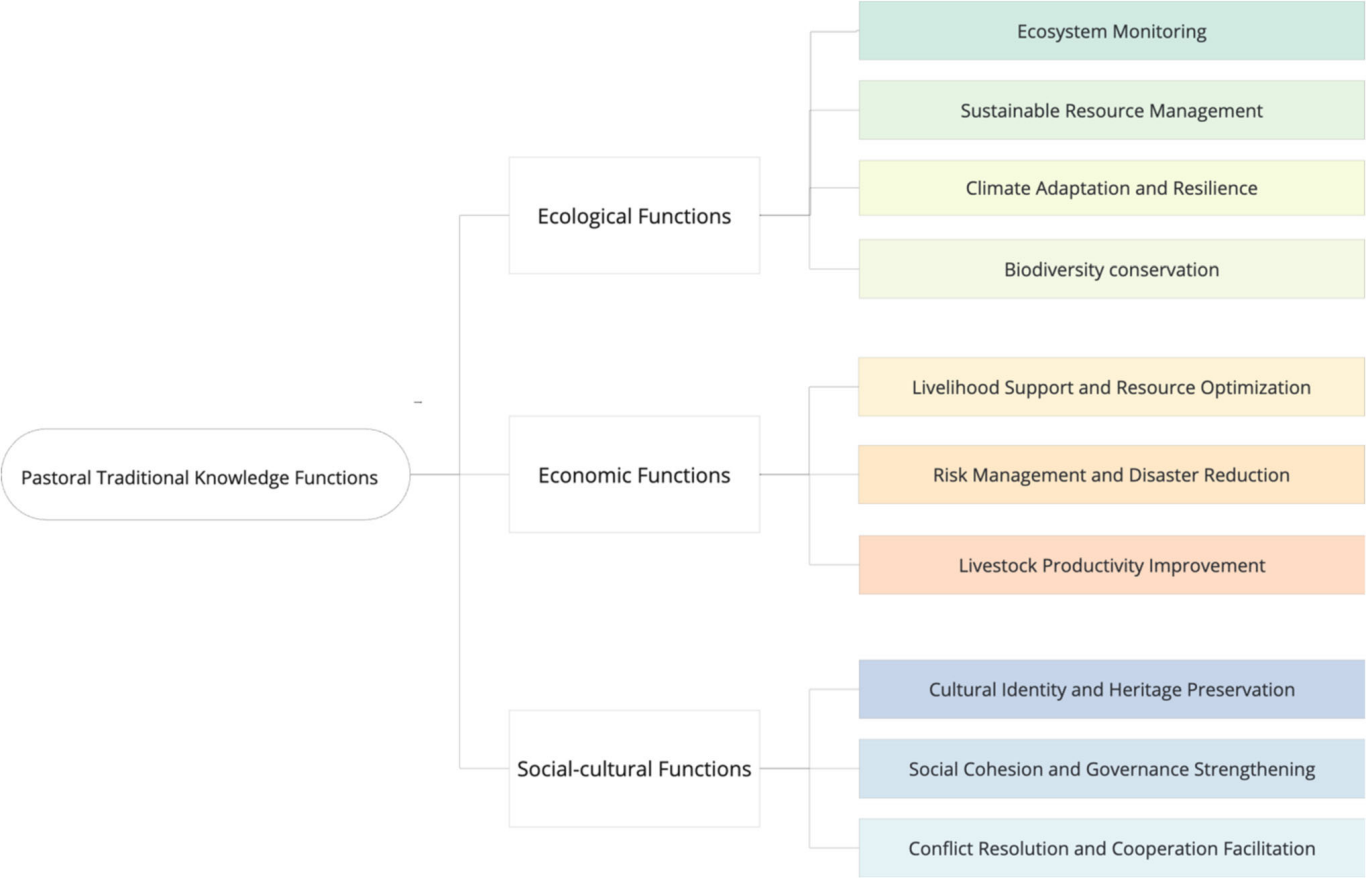
Hierarchical classification tree presenting a classification of PTK functions

Our analysis suggests that PTK covers different ecological, economic, and social–cultural functions in pastoral systems (Fig. 2). PTK ecological functions refer to the roles and significance of PTK in interacting with and sustaining nature. PTK ecological functions include various subfunctions such as monitoring ecosystem health, preventing unsustainable resource use, predicting weather and climate variations, and maintaining biological diversity in the ecosystem.

Within the dataset analyzed, a total of 252 citations are applied to support ecological functions. Upon examining the distribution of these citations among different knowledge domains, it is apparent that the domain of herd mobility practice is particularly significant. Thirty-one percent of the citations (n = 78) that play ecological functions belong to herd mobility practice. For example, some communities use herd mobility to allow pasture regeneration, while others mainly use it to sustainably manage natural resources (Stammler-Gossmann 2010).

Climate and weather-related knowledge is also prominently associated with ecological functions. Twenty-two percent of the citations which play ecological functions are found in this domain. For example, in various communities, weather forecasting knowledge was used to adapt to climate variations (Radeny et al. 2019; Inman et al. 2020). Finally, landscape-related knowledge makes up 13% (n = 32) of citations that play ecological functions. Controlled fire practice, for example, was used in many pastoral communities to encourage grass growth (Bollig and Österle 2008).

**Fig. 3:**
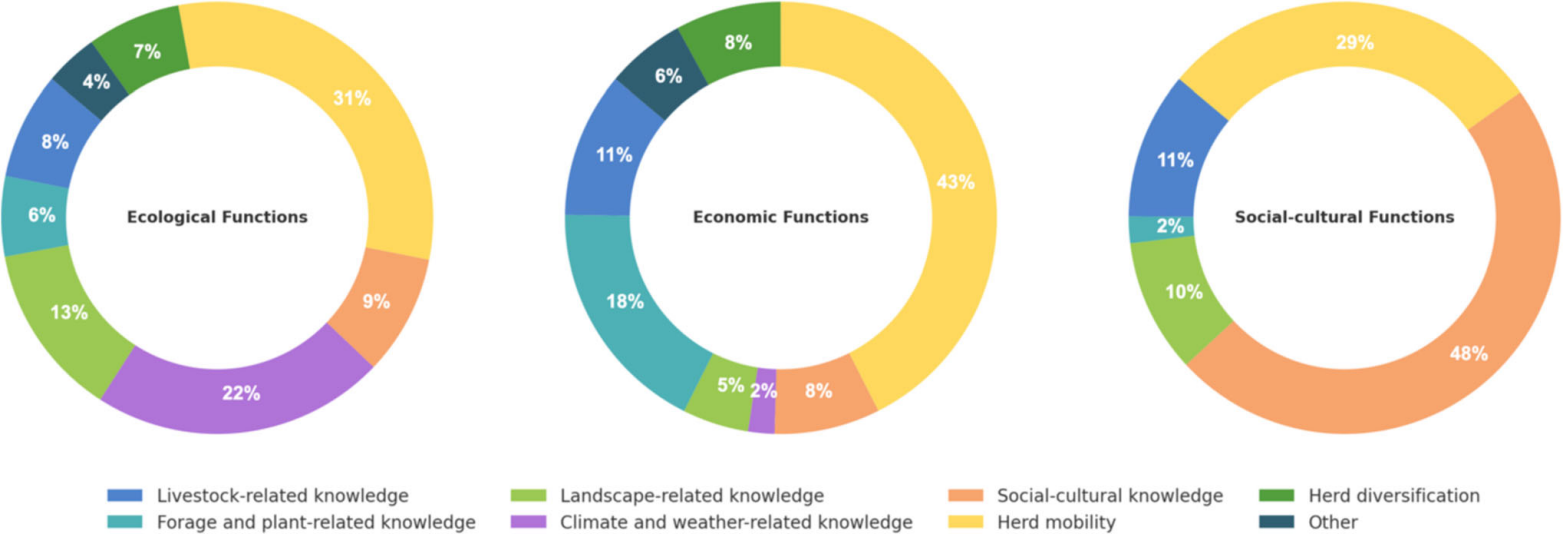
Pie charts presenting the share of PTK domains associated with ecological, economic, and socio-cultural functions, ordered by citation frequency from the most cited (ecological) to the least (socio-cultural)

PTK’s economic functions refer to PTK’s role in enhancing the efficiency and sustainability of pastoralists’ livelihoods. We identified three different subfunctions in this group: utilizing limited resources effectively, mitigating the impacts of natural disasters, and improving livestock productivity and health. Among all the recorded citations, there are 139 of them which play economic functions among pastoral communities. The domain of herd mobility emerges as the most prominent within this economic category, with 43% (59 citations) attributed to the herd mobility practice. Pastoralists in Greece, for instance, maintain herd health and maximize herd productivity by moving them to different grazing areas to access fresh vegetation (Siasiou et al. 2020). Although forage/plant-related knowledge is not prominently featured for its ecological function, this knowledge domain made up 18% (26 citations) of PTK economic functions (Fig. 3). For example, the Rabari pastoralists in India utilize *Prosopis juliflora* leaves as medicine for herd skin infections to mitigate the loss of livestock productivity due to untreated ailments (Duenn et al. 2017). Livestock-related knowledge accounts for 11% (16 citations) of the economic functional group. For example, Sámi and Tibetan herders have the knowledge and skills to evaluate the clinical signs of their herd to prevent disease (Haynes and Yang 2012; Riseth et al. 2020).

The social–cultural functions of PTK contribute to the maintenance of pastoral communities’ cultural integrity and social structures. This functional characteristic stresses PTK’s role in preserving traditional culture but also in enhancing the social bond of the community and promoting cooperation. Among the recorded citations, 59 citations are documented as fulfilling social–cultural functions. Social–cultural functions draw in social–cultural knowledge, which forms nearly half of this functional category (48%, 29 citations). However, it is interesting to note that livestock-related knowledge, accounting for 11% of all social–cultural functions, and landscape-related knowledge, representing 10%, also contribute meaningfully to the social–cultural fabric. For example, the *tsamdro* management practice of Brokpa pastoralists in Bhutan, which involves the use of dry-stack stone walls and wooden fences to manage livestock, reduces grazing conflicts, thus playing a crucial role in fostering social harmony within these communities (Wangdi and Norbu 2018). Additionally, the deliberate choice by pastoralists to favor traditional livestock breeds is a way to sustain local pastoral culture and identity in many communities (Fernández-Giménez 2015).

**Fig. 4:**
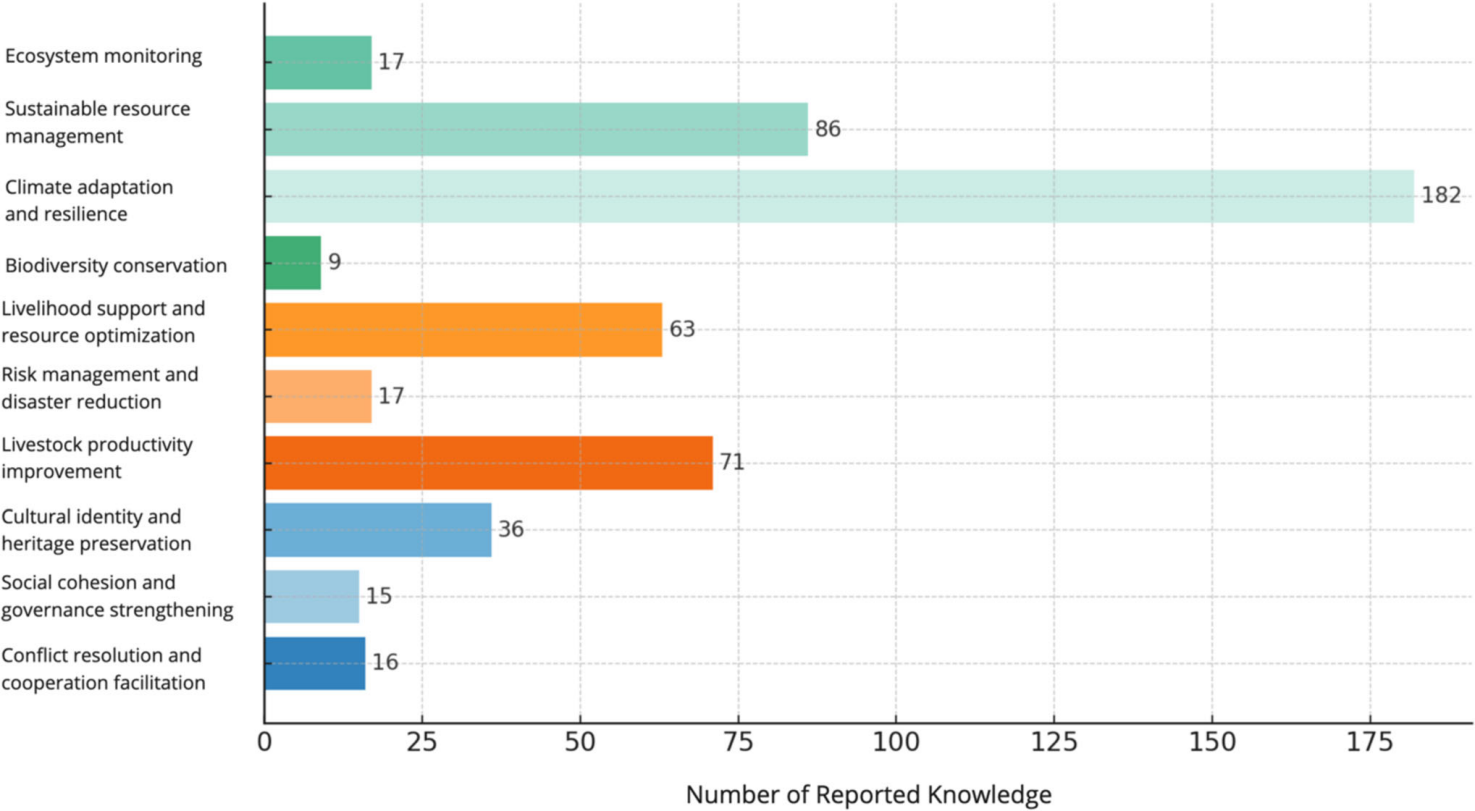
Frequency of mention of different ecological, economic, and socio-cultural subfunctions of PTK

We further analyzed the data to better understand the weight of different subfunctions within the three main functions (Fig. 4). We did so by categorizing the 252 citations that play ecological functions into different sub-ecological groups. It is important to note that while some pieces of knowledge were unique to a single function, others had multiple functions. Likewise, the 139 citations that play economic functions and the 59 citations that play social–cultural functions were also further classified into specific subfunctions. The ecological functions of PTK stand out as the most common, with over half (58%) of the recorded subfunctions addressing four ecological functions: ecosystem monitoring, sustainable resource use, climate adaptation and resilience, and biodiversity conservation. Within ecological functions, climate adaptation and resilience is the most often cited subfunction, comprising 35% of all recorded citations. The subfunction of sustainable resource management represents 17% of the citations. Within economic functions, enhancing livestock productivity (14%) and livelihood support (12%) are two subfunctions most often cited. Overall, the social–cultural functions of PTK are the less often cited. Within those, cultural preservation (7%) is emphasized to a larger extent than some ecological and economic functions, including ecosystem monitoring and risk management.

### Multifunctional characteristic of PTK domains

**Fig. 5:**
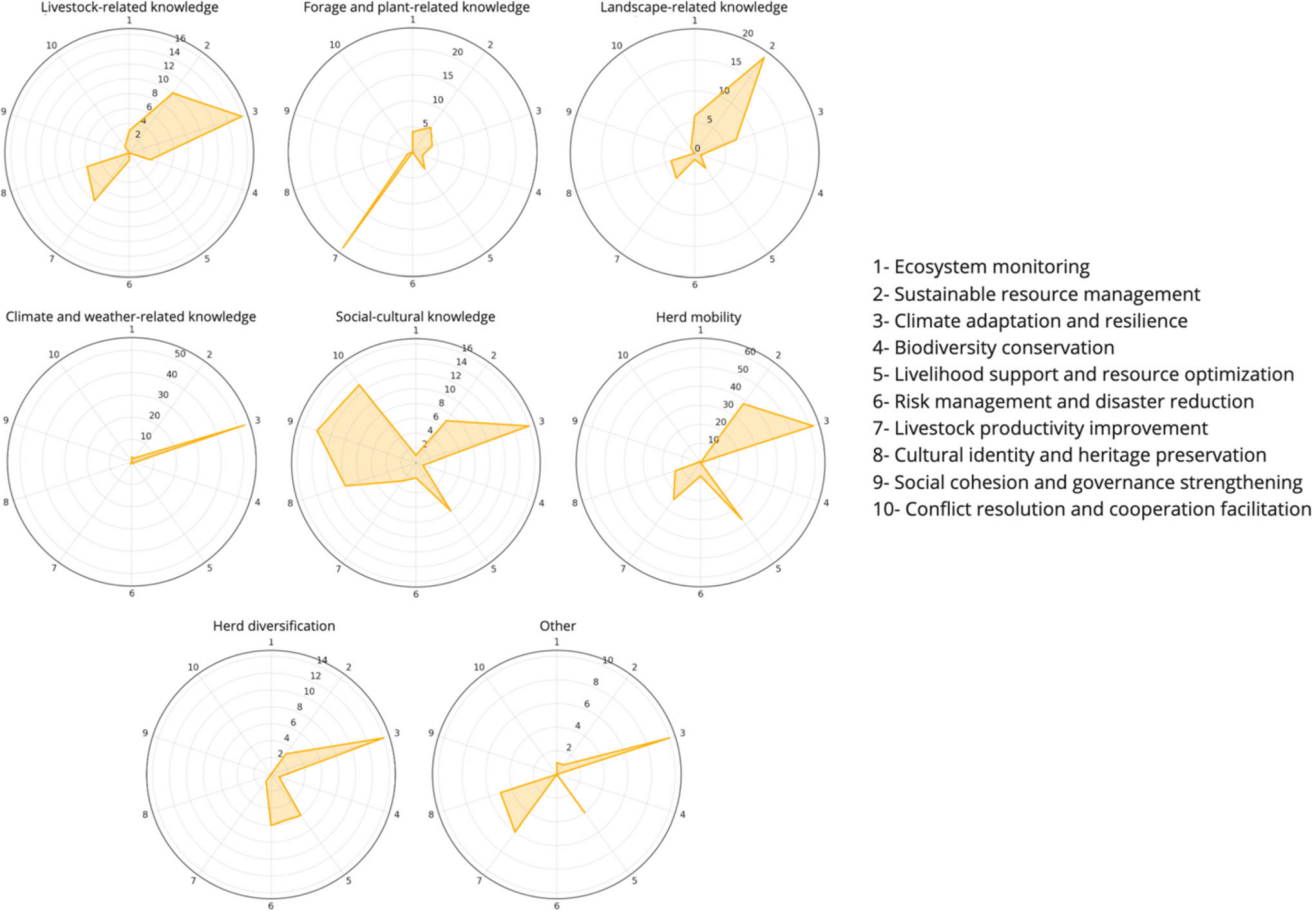
Contribution of different knowledge domains to different subfunctions

The visual representation of how different knowledge domains contribute to various functions reveals that most domains are connected to distinct functions (Fig. 5). For instance, social–cultural knowledge covers ten types of subfunctions, ranging from climate adaptation and resilience to social cohesion and community governance. Climate/weather-related knowledge (29%) significantly contributes to climate adaptation and resilience. Interestingly, livestock-related knowledge, which one might expect to predominantly impact areas directly related to herd management, such as productivity, in fact, shows a diverse range of functions. Indeed, while livestock-related knowledge contributes to herd productivity (11%), slightly more citation is observed in cultural identity preservation (17%), and sustainable resource management (12%). Another notable aspect is that social–cultural knowledge not only fulfills important functions in cultural identity, social cohesion, and conflict resolution but also extends to ecological and economic subfunctions, such as contributing to climate adaptation and resilience (9%) and risk management (13%).

### Common functions across PTK domains

In our comprehensive analysis of PTK, a pattern of common functions among various knowledge domains emerged. Figure 5 suggests that all the knowledge domains examined collectively contribute to sustainable resource management, and climate adaptation and resilience functions. Specifically, herd mobility practice, landscape-related knowledge, and livestock-related knowledge are the most recorded as contributing to the sustainable management of resources. Climate/weather-related knowledge, social–cultural knowledge, and mobility practice are often cited as serving the climate adaptation and resilience subfunction, jointly addressing the challenges of environmental changes. For ecosystem monitoring, landscape-related knowledge emerged as the key contributor.

In terms of knowledge domains contributing to economic subfunctions, all the knowledge domains were found to jointly contribute to improving livestock productivity. Furthermore, herd diversification and herd mobility practices were often intertwined in addressing risk management.

Culturally, almost all the knowledge domains contributed to the preservation of cultural identity and heritage. Although social–cultural knowledge played a dominant function in improving social cohesion and community governance, some case studies reported that herd mobility could serve the same purpose. For example, Fulani pastoralists have effectively minimized competition for scarce resources like water and grazing lands—common sources of conflict—by strategically relocating their herds to various locations (Bassett 1986, as cited in Wario et al. 2016). Social–cultural-related knowledge, followed by livestock-related and landscape-related knowledge, contributes the most to avoiding or solving conflicts and enhancing cooperation.

## Discussion

Our review is the first to use a functional lens to analyze traditional knowledge systematically. This approach offers a novel perspective to understand traditional knowledge functional characteristics. We focus on the analysis of different knowledge domains within traditional knowledge systems and the different functions and subfunctions each knowledge domain plays to support and nurture communities. Extensive literature suggests that the resilience of traditional communities is deeply rooted in their adaptive knowledge systems. These systems are responsive to environmental changes and play a significant role in sustainable ecosystem management (Berkes et al. 2000; Reyes‐García et al. 2018). Our review delves into how traditional knowledge, particularly PTK, has historically and contemporarily contributed to the strength and adaptability of the community. We find that PTK serves diverse functions within pastoral communities, illustrating a complex interplay between various knowledge domains and their respective functions.

### Domains of pastoral traditional knowledge

Research has suggested that pastoral communities are remarkably adaptable worldwide, for which they rely on their rich and diverse knowledge (Hausner et al. 2020; Tugjamba et al. 2021). In accordance with previous review work (e.g., Turner and Schlecht 2019), our findings show that data about PTK in scientific publications are dominated by mobility practice and climate/weather-related knowledge. Mobility, a key practice, is widely practiced by pastoralists globally. It is mentioned in 124 of the 152 case studies we analyzed. Mobility takes diverse forms, from the movement of the Qashqai nomads in Iran (Saboohi et al. 2018) to the transhumance practices among the Hutsul communities in Ukraine (Fontana et al. 2022) or the *otor* movement among the Mongolian herders (Zhang et al. 2013).

The increasing research interest in how pastoral communities manage climate variability and change (Ayele et al. 2020) is mirrored in the identification of climate/weather-related knowledge as the second most reported knowledge domain in our review, featured in 63 cases. Specific instances of climate/weather-related knowledge include knowledge of weather forecasting (Radeny et al. 2019) or observations of climate-related changes (Klein et al. 2014). In response to climate change, weather and climate knowledge empowers pastoralists to adapt to climate variability, a theme supported by many studies (Oteros-Rozas et al. 2013; Inman et al. 2020; Camacho-Villa et al. 2021).

However, our study also reveals significant imbalances in the focus of existing case studies regarding knowledge domains. The fundamental knowledge of pastoralism globally, such as livestock-related (10% of the cases), forage/plant, and landscape-related knowledge are significantly underrepresented. It is difficult to believe that pastoral communities do not possess this knowledge, as it is central to their way of life and essential for managing their environments. Additionally, we found that more than half of the case studies (55%) investigated only one or two knowledge domains. This underrepresentation is problematic because it fails to capture the full complexity and interconnectedness of the pastoral knowledge system. Global research on traditional pastoral knowledge shows that pastoralists across diverse regions use a complex and common set of principles, including forage/plant, landscape, and livestock knowledge to manage resources efficiently and sustain their livelihoods (Sharifian et al. 2023). By focusing narrowly on certain aspects, research risks oversimplifying the holistic strategies that pastoralists employ. Pastoralists do not view these domains in isolation; rather, they integrate multiple domains to adapt to environmental uncertainties and ensure the sustainability of their resources. Thus, we argue that this narrow focus and fragmented approach risks presenting an incomplete or even distorted understanding of PTK. It fails to capture the holistic strategies pastoralists use to manage uncertainty and ensure the sustainability of their resources.

### Functions of pastoral traditional knowledge

Moving from the specific domains of PTK to its broader implications, PTK exhibits diverse functions, playing ecological, economic, and socio-cultural roles. In our database, the most common ecological subfunction of PTK is climate adaptation and resilience, which appears more frequently than the sum of the rest of the ecological subfunctions. This prominence likely stems from pastoralists’ direct experience with climate variability, such as droughts, floods, and shifting seasonal patterns, underscoring their adeptness at navigating and mitigating the adverse effects of weather variability, and potentially of climate change (e.g., Ifejika Speranza 2010; Ahmed et al. 2023). The review by Arjjumend’s (2018) further underscores how pastoralists’ knowledge system enables them to cope with environmental unpredictability and variability through a variety of strategies, such as diversification and mobility.

Regarding the economic aspect, PTK is mainly mentioned for enhancing herd productivity, and livelihood support and resource optimization. The findings show that forage/plant-related knowledge and mobility practice are the core for maintaining herd health and productivity. This aligns with the findings of Launchbaugh (2020), who highlighted that livestock could benefit from mobile grazing behavior by taking a variety of forage with different nutritional qualities. Therefore, having knowledge about forage/plant identification and utilizing herd mobility is vital for maintaining herd health and productivity. Additionally, rangeland resources are often limited and can vary spatially and temporally due to factors like rainfall patterns, topography, and soil types (Godde et al. 2020). In such environments, the ability to optimize resource use becomes crucial for maintaining herd productivity. This strongly reinforces the significance of our findings.

In terms of social–cultural functions, we found that livestock-related knowledge and landscape-related knowledge play significant roles in preserving the cultural identity and heritage of the pastoral communities. Similar findings are also presented in previous work (Russell and Ward 2016; Ahozonlin and Dossa 2020). For example, Gandini and Villa (2003) argue local breeds should be regarded as cultural properties due to their contribution to the preservation of ancient local traditions. Furthermore, the landscapes that pastoralists inhabit and manage are imbued with cultural significance. Managing and preserving these landscapes, therefore, becomes an act of cultural heritage conservation. In certain communities, specific practices in landscape management, like controlled burning, are important parts of their culture (Fernández-Giménez 2015).

Building on the understanding of these varied subfunctions, our findings suggest that each domain of PTK serves multiple ecologic, economic, and socio-cultural functions. For instance, in Peru, mobility helps mitigate overgrazing and facilitates natural regeneration; in Inner Mongolia, it preserves traditional nomadic culture; and among the Maasai herders in Kenya, it enhances herd productivity and aids adaptation to drought conditions (Huang et al. 2009; Sundstrom et al. 2012; López-i-Gelats et al. 2015). The significance of multifunctional characteristic within the knowledge system is profound. It enhances community stability by equipping them with a diverse set of strategies to deal with uncertainties and ensures that knowledge itself remains pertinent and flexible, capable of adjusting to evolving challenges. As some case studies show, even when a community is faced with constraints such as privatization, mobility adapts to fulfill other important functions, such as land preservation. For example, in response to land degradation and the fragmentation of pastures in Inner Mongolia, herders increasingly adopt the traditional mobility strategy, enabling rotational use of pastures to mitigate overgrazing and promote grassland ecosystem regeneration (Xie and Li 2012). This gives reason to believe that the multifunctional characteristic of traditional knowledge systems contributes to their continued relevance and transmission. Therefore, future studies could examine this relation more directly. Additionally, there is a significant opportunity for future studies to explore how these functions evolve and adapt over time, particularly whether knowledge domains develop new functions in response to environmental and social changes.

Expanding on the finding of multifunctional characteristic of each domain, another finding that deserves attention is the substantial common functions present across knowledge domains. For example, all eight knowledge domains contribute to sustainable resource management, and climate adaptation and resilience, albeit with varying degrees of contributions. Similarly, all knowledge domains contribute to enhancing livestock productivity, an economic subfunction. Compared with these two functional groups, social–cultural functions present fewer overlaps across different knowledge domains, indicating that these functions may be more vulnerable to disruptions or change as they rely less on functional overlaps across multiple knowledge domains for their fulfillment. The idea that communities utilize alternative knowledge to achieve similar outcomes due to various challenges is discussed in the existing literature. For instance, Gauer et al. (2021) explore how indigenous communities adapt their knowledge in response to environmental changes, employing alternative strategies when certain knowledge becomes impractical or ineffective. Similarly, Berkes et al. (2000) highlight how environmental and social changes force communities to substitute or modify traditional practices, preserving the functionality of their knowledge systems despite new challenges. Drawing on these findings, we propose that future studies could interpret this interplay and synergy as a mode of strengthening traditional knowledge systems. We hypothesize that the common functions identified across different PTK domains allow pastoral communities to approach challenges such as climate change from various angles, thereby increasing the likelihood of finding a more effective solution.

Given the diverse range and complex interplay of ecological, economic, and socio-cultural functions within PTK, there is a need for adopting an interdisciplinary approach. As Berkes et al. (2000) insightfully point out, researchers who lack expertise in ecology may not recognize the ecological significance of traditional knowledge. Conversely, ecologists may not fully capture the social and cultural significance of traditional knowledge. Therefore, by incorporating perspectives from various fields, future studies can achieve a more holistic understanding of traditional knowledge systems and their functions. Additionally, there is a need for future studies to involve and collaborate directly with local communities. In this way, researchers can ensure that their work captures the full depth and interconnectedness of PTK while respecting and valuing the perspectives and lived experiences of these communities.

## Supplementary Information

Below is the link to the electronic supplementary material.

Supplementary file1 (PDF 294 kb)

## Data Availability

The data that support the findings of this study will shortly be openly available in Figshare repository.
