# Supplementary material for: Exploring the dynamic functions of pastoral traditional knowledge
Source: Ambio. 2025 Feb 1;54(6):932–46. doi: 10.1007/s13280-025-02131-x (PMC12055689; doi:10.1007/s13280-025-02131-x)
Supplement: Supplementary file 1 — Supplementary file1 (PDF 294 kb) [file 13280_2025_2131_MOESM1_ESM.pdf]

Ambio

Supplementary Information

This supplementary information has not been peer reviewed.

Title: **Exploring the Dynamic Functions of Pastoral Traditional Knowledge**

Supplementary Information A

Table S1. List of publications included in the systematic literature review

Paper ID No.	References
11	Ghai, R. (2021). Understanding ‘culture’ of pastoralism and ‘modern development’ in thar: Muslim pastoralists of north-West Rajasthan, India. *Pastoralism, 11*(1). https://doi.org/10.1186/s13570-020-00190-1
12	Wang X., and Liao C., Brandhorst S.M., Clark P.E. (2022). Sedentarization as an adaptation to socio-environmental changes? Everyday herding practices in pastoralist communities in southern Ethiopia. *Ecology and Society*, https://doi.org/10.5751/ES-13503-270339
15	Carmona R. (2022). Resilience Requires Change: Assessing Pehuenche Responses to Climate Change Impacts in Southern Chile. *Environmental Justice*, https://doi.org/10.1089/env.2021.0044
17	Vargas-López S., and Bustamante-González A., Ramírez-Bribiesca J.E., Torres-Hernández G., Larbi A., Maldonado-Jáquez J.A., López-Tecpoyotl Z.G. (2022). Rescue and participatory conservation of Creole goats in the agro-silvopastoral systems of the Mountains of Guerrero, Mexico. *Revista de la Facultad de Ciencias Agrarias*, https://doi.org/10.48162/rev.39.074
21	Malhotra A., and Nandigama S., Bhattacharya K.S. (2022). Puhals: Outlining the Dynamics of Labour and Hired Herding among the Gaddi Pastoralists of India. *Pastoralism*, https://doi.org/10.1186/s13570-022-00237-5
26	Fernández-Giménez M.E., and Ravera F., Oteros-Rozas E. (2022). The invisible thread: women as tradition keepers and change agents in Spanish pastoral social-ecological systems. *Ecology and Society*, https://doi.org/10.5751/ES-12794-270204

27	Kaoga J., and Olago D., Ouma G., Ouma G., Onono J. (2021). The evolving cultural values and their implications on the Maasai Pastoralists, Kajiado County, Kenya. *Scientific African*, https://doi.org/10.1016/j.sciaf.2021.e00881
31	Dejene A., and Yetebarek H. (2022). The Relevance and Practices of Indigenous Weather Forecasting Knowledge among the Gabra Pastoralists of Southern Ethiopia. *Journal of Agriculture and Environment for International Development*, https://doi.org/10.36253/jaeid-12295
36	Napogbong L.A., and Domapielle M.K., Derbile E.K. (2021). Indigenous knowledge and community-based risk assessment of climate change among the Fulani Herder Community of Kpongu, North-Western Ghana. *Journal of Water and Climate Change*, https://doi.org/10.2166/wcc.2020.236
39	Reid-Shaw I., and Jargalsaihan A., Reid R.S., Jamsranjav C., Fernández-Giménez M.E. (2021). Social-Ecological Change on the Mongolian Steppe: Herder Perceptions of Causes, Impacts, and Adaptive Strategies. *Human Ecology*, https://doi.org/10.1007/s10745-021-00256-7
40	Ghazali S., and Azadi H., Janečková K., Sklenička P., Kurban A., Cakir S. (2021). Indigenous knowledge about climate change and sustainability of nomadic livelihoods: understanding adaptability coping strategies. *Environment, Development and Sustainability*, https://doi.org/10.1007/s10668-021-01332-0
42	Holand Ø., and Mäki-Tanila A., Kvalnes T., Muuttoranta K., Paoli A., Pietarinen J., Weladji R.B., Åhman B. (2022). The productive herd: Past, present and perspectives. *Reindeer Husbandry and Global Environmental Change: Pastoralism in Fennoscandia*, https://doi.org/10.4324/9781003118565-15
43	Miara M.D., and Negadi M., Tabak S., Bendif H., Dahmani W., Ait Hammou M., Sahnoun T., Snorek J., Porcher V., Reyes-García V., Teixidor-Toneu I. (2022). Climate Change Impacts Can Be Differentially Perceived Across Time Scales: A Study Among the Tuareg of the Algerian Sahara. *GeoHealth*, https://doi.org/10.1029/2022GH000620
44	Fernandez-Gimenez, and ME, El Aich, A, El Aouni, O, Adrane, I, El Aayadi, S (2021). Ilemchane Transhumant Pastoralists' Traditional Ecological Knowledge and Adaptive Strategies: Continuity and

	Change in Morocco's High Atlas Mountains. *MOUNTAIN RESEARCH AND DEVELOPMENT*, https://doi.org/10.1659/MRD-JOURNAL-D-21-00028.1
47	Lavrillier A., and Gabyshev S. (2021). An Indigenous science of the climate change impacts on landscape topography in Siberia. *Ambio*, https://doi.org/10.1007/s13280-020-01467-w
49	Tugjamba N., and Walkerden G. (2021). Traditional and modern ecosystem services thinking in nomadic Mongolia: Framing differences, common concerns, and ways forward. *Ecosystem Services*, https://doi.org/10.1016/j.ecoser.2021.101360
54	Landauer, and M, Rasmus, S, Forbes, BC (2021). What drives reindeer management in Finland towards social and ecological tipping points?. *REGIONAL ENVIRONMENTAL CHANGE*, https://doi.org/10.1007/s10113-021-01757-3
55	Ulziibaatar M., and Matsui K. (2021). Herders' perceptions about rangeland degradation and herd management: A case among traditional and non-traditional herders in khentii province of mongolia. *Sustainability (Switzerland)*, https://doi.org/10.3390/su13147896
57	Lelenguyah G.L., and Nyangito M.M., Wasonga O.V., Bett R.C. (2022). Herders' Perspectives on Climate Variability and Livestock Diseases Trends in the Semiarid Rangelands of Northern Kenya. *International Journal of Climate Change: Impacts and Responses*, https://doi.org/10.18848/1835-7156/CGP/v15i02/69-88
60	Saboohi R., and Barani H., Khodagholi M., Sarvestani A.A., Tahmasebi A., Feuer H.N. (2022). Aligning Trends in Climatic Parameters and Nomads' Indigenous Knowledge about Climate Change in Central Iran (Case Study: Semirom town). *Weather, Climate, and Society*, https://doi.org/10.1175/WCAS-D-21-0041.1
61	Arzamendia Y., and Rojo V., González N.M., Baldo J.L., Zamar M.I., Lamas H.E., Vilá B.L. (2021). The Puna Pastoralist System: A Coproduced Landscape in the Central Andes. *Mountain Research and Development*, https://doi.org/10.1659/MRD-JOURNAL-D-21-00023.1
67	Wang G., and Yang L., Liu M., Li Z., He S., Min Q. (2021). The Role of Local Knowledge in the Risk Management of Extreme Climates in Local Communities: A Case Study in a Nomadic NIAHS Site;

	[地方知识在社区的极端气候风险管理中的角色--基于游牧类重要农业文化遗产的研究]. *Journal of Resources and Ecology*, https://doi.org/10.5814/j.issn.1674-764x.2021.04.011
70	Tugjamba N., and Walkerden G., Miller F. (2021). Climate change impacts on nomadic herders' livelihoods and pastureland ecosystems: a case study from Northeast Mongolia. *Regional Environmental Change*, https://doi.org/10.1007/s10113-021-01829-4
81	Dabasso B.G., and Makokha A.O., Onyango A.N., Mathara J.M. (2022). Beyond nutrition: social–cultural values of meat and meat products among the Borana people of Northern Kenya. *Journal of Ethnic Foods*, https://doi.org/10.1186/s42779-022-00160-5
89	Fontefrancesco M.F., and Zocchi D.M., Cevasco R., Dossche R., Abidullah S., Pieroni A. (2022). Crumbotti and rose petals in a ghost mountain valley: foraging, landscape, and their transformations in the upper Borbera Valley, NW Italy. *Journal of Ethnobiology and Ethnomedicine*, https://doi.org/10.1186/s13002-022-00535-7
92	Konnov A., and Khmelnitskaya Y., Dugina M., Borzenko T., Tysiachniouk M.S. (2022). Traditional Livelihood, Unstable Environment: Adaptation of Traditional Fishing and Reindeer Herding to Environmental Change in the Russian Arctic. *Sustainability (Switzerland)*, https://doi.org/10.3390/su141912640
94	Fontana N.M., and Pasailiuk M.V., Pohribnyi O. (2022). Traditional ecological knowledge to traditional foods: The path to maintaining food sovereignty in Hutsulshchyna. *Frontiers in Sustainable Food Systems*, https://doi.org/10.3389/fsufs.2022.720757
107	Nkuba M., and Chanda R., Mmopelwa G., Kato E., Mangheni M.N., Lesolle D. (2019). The effect of climate information in pastoralists' adaptation to climate change: A case study of Rwenzori region, Western Uganda. *International Journal of Climate Change Strategies and Management*, https://doi.org/10.1108/IJCCSM-10-2018-0073
116	Tugjamba N., and Walkerden G., Miller F. (2021). Adaptation strategies of nomadic herders in northeast Mongolia: climate, globalisation and traditional knowledge. *Local Environment*, https://doi.org/10.1080/13549839.2021.1891032

117	Nyima Y., and Hopping K.A. (2019). Tibetan Lake Expansion from a Pastoral Perspective: Local Observations and Coping Strategies for a Changing Environment. *Society and Natural Resources*, https://doi.org/10.1080/08941920.2019.1590667
130	Solano-Hernandez A., and Bruzzone O., Groot J., Laborda L., Martínez A., Tittonell P., Easdale M.H. (2020). Convergence between satellite information and farmers' perception of drought in rangelands of North-West Patagonia, Argentina. *Land Use Policy*, https://doi.org/10.1016/j.landusepol.2020.104726
131	Rayamajhi N., and Manandhar B. (2020). Impact of Climate Change and Adaptation Measures on Transhumance Herding System in Gatlang, Rasuwa. *Air, Soil and Water Research*, https://doi.org/10.1177/1178622120951173
134	Liberman M. (2021). Climate Change, Wetland Management and Alpaca Pastoralism in the Bolivian High Andes Mountains. *Geobotany Studies*, https://doi.org/10.1007/978-3-030-74950-7_5
135	Snaibi W., and Mezrhab A. (2021). Livestock Breeders' Adaptation to Climate Variability and Change in Morocco's Arid Rangelands. *African Handbook of Climate Change Adaptation: With 610 Figures and 361 Tables*, https://doi.org/10.1007/978-3-030-45106-6_18
142	Ngute A.S.K., and Marchant R., Cuni-Sanchez A. (2021). Climate Change, Perceptions, and Adaptation Responses Among Farmers and Pastoralists in the Cameroon Highlands. *Handbook of Climate Change Management: Research, Leadership, Transformation*, https://doi.org/10.1007/978-3-030-57281-5_311
143	Carrer F., and Walsh K., Mocci F. (2020). Ecology, Economy, and Upland Landscapes: Socio-Ecological Dynamics in the Alps during the Transition to Modernity. *Human Ecology*, https://doi.org/10.1007/s10745-020-00130-y
153	Arora A. (2020). Values, environmental vulnerabilities, and implications on adaptation: Evidence from an indigenous Raika community in Rajasthan, India. *International Journal of Population Studies*, https://doi.org/10.18063/ijps.v6i1.1066

155	Nursey-Bray M., and Palmer R., Stuart A., Arbon V., Rigney L.-I. (2020). Scale, colonisation and adapting to climate change: Insights from the Arabana people, South Australia. *Geoforum*, https://doi.org/10.1016/j.geoforum.2020.05.021
166	Zhang Q., and Cui F., Dai L., Feng B., Lu Y., Tang H. (2019). Pastoralists' perception of and adaptation strategies for climate change: associations with observed climate variability. *Natural Hazards*, https://doi.org/10.1007/s11069-019-03620-5
171	Inman E.N., and Hobbs R.J., Tsvuura Z. (2020). No safety net in the face of climate change: The case of pastoralists in Kunene Region, Namibia. *PLoS ONE*, https://doi.org/10.1371/journal.pone.0238982
172	Korir J.C. (2019). Level of awareness about climate change among the pastoral community. *Environment and Ecology Research*, https://doi.org/10.13189/eer.2019.070401
174	Postigo J.C. (2021). The role of social institutions in indigenous Andean Pastoralists' adaptation to climate-related water hazards. *Climate and Development*, https://doi.org/10.1080/17565529.2020.1850409
176	Ahozonlin M.C., and Dossa L.H. (2020). Diversity and resilience to socio-ecological changes of smallholder lagune cattle farming systems of Benin. *Sustainability (Switzerland)*, https://doi.org/10.3390/su12187616
177	Riseth J.Å., and Tømmervik H., Tryland M. (2020). Spreading or gathering? Can traditional knowledge be a resource to tackle reindeer diseases associated with climate change?. *International Journal of Environmental Research and Public Health*, https://doi.org/10.3390/ijerph17166002
178	Zhe Y., and Cili J., Mace R.H., Juan D., Pengpeng B., Guozhen D. (2019). Adaptive Strategies Adopted by Herders Against the Decollectivization of Rangeland in the Qinghai-Tibetan Plateau in China. *Mountain Research and Development*, https://doi.org/10.1659/MRD-JOURNAL-D-19-00034.1
184	Axelsson-Linkowski W., and Fjellström A.-M., Sandström C., Westin A., Östlund L., Moen J. (2020). Shifting Strategies between Generations in Sami Reindeer Husbandry: the Challenges of Maintaining Traditions while Adapting to a Changing Context. *Human Ecology*, https://doi.org/10.1007/s10745-020-00171-3

199	Saboohi R., and Barani H., Khodagholi M., Sarvestani A.A., Tahmasebi A. (2019). Nomads' indigenous knowledge and their adaptation to climate changes in Semirom City in Central Iran. *Theoretical and Applied Climatology*, https://doi.org/10.1007/s00704-018-2665-4
200	Wangui E.E., and Smucker T.A. (2018). Gendered opportunities and constraints to scaling up: a case study of spontaneous adaptation in a pastoralist community in Mwanga District, Tanzania. *Climate and Development*, https://doi.org/10.1080/17565529.2017.1301867
202	Dong S. (2017). Himalayan Grasslands: Indigenous Knowledge and Institutions for Social Innovation. *Environmental Sustainability from the Himalayas to the Oceans: Struggles and Innovations in China and India*, https://doi.org/10.1007/978-3-319-44037-8_5
205	Tilahun M., and Angassa A., Abebe A. (2017). Community-based knowledge towards rangeland condition, climate change, and adaptation strategies: the case of Afar pastoralists. *Ecological Processes*, https://doi.org/10.1186/s13717-017-0094-4
215	Lavrillier A., and Gabyshev S. (2018). An emic science of climate. Reindeer Evenki environmental knowledge and the notion of an "extreme process"; [Une science émique du climat. Le savoir environnemental des évenks et la notion de « processus extrême »]. *Etudes Mongoles et Siberiennes, Centrasiatiques et Tibetaines*, https://doi.org/10.4000/emscat.3280
216	Aryal S., and Cockfield G., Maraseni T.N. (2018). Globalisation and traditional social-ecological systems: Understanding impacts of tourism and labour migration to the transhumance systems in the Himalayas. *Environmental Development*, https://doi.org/10.1016/j.envdev.2017.09.001
218	Soma T., and Schlecht E. (2018). The relevance of herders' local ecological knowledge on coping with livestock losses during harsh winters in western Mongolia. *Pastoralism*, https://doi.org/10.1186/s13570-017-0108-y
219	Volpato G., and Di Nardo A. (2017). The role of Nucularia perrinii Batt. (Chenopodiaceae) in the camel-based Sahrawi social-ecological system. *Journal of Ethnobiology and Ethnomedicine*, https://doi.org/10.1186/s13002-017-0141-3

224	Houessou S.O., and Dossa L.H., Diogo R.V.C., Houinato M., Buerkert A., Schlecht E. (2019). Change and continuity in traditional cattle farming systems of West African Coast countries: A case study from Benin. *Agricultural Systems*, https://doi.org/10.1016/j.agsy.2018.11.003
228	Basupi L.V., and Quinn C.H., Dougill A.J. (2017). Historical perspectives on pastoralism and land tenure transformation in Ngamiland, Botswana: What are the policy and institutional lessons?. *Pastoralism*, https://doi.org/10.1186/s13570-017-0093-1
230	Marshall K., and Mtimet N., Wanyoike F., Ndiwa N., Ghebremariam H., Mugunieri L., Costagli R. (2016). Traditional livestock breeding practices of men and women Somali pastoralists: trait preferences and selection of breeding animals. *Journal of Animal Breeding and Genetics*, https://doi.org/10.1111/jbg.12223
233	Matsa M. (2018). Climate Change and Tonga Community Development: Thinking from the Periphery. *Human and Environmental Security in the Era of Global Risks: Perspectives from Africa, Asia and the Pacific Islands*, https://doi.org/10.1007/978-3-319-92828-9_16
241	Singh R.K., and Sureja A.K., Maiti S., Tsering D. (2018). Grazing and rangeland management: Trans-human adaptations by Brokpa community in fragile ecosystems of Arunachal Pradesh. *Indian Journal of Traditional Knowledge*, https://doi.org/0
242	Kagunyu A., and Wandibba S., Wanjohi J.G. (2016). The use of indigenous climate forecasting methods by the pastoralists of Northern Kenya. *Pastoralism*, https://doi.org/10.1186/s13570-016-0054-0
245	Ansari-Renani H.R. (2016). An investigation of organic sheep and goat production by nomad pastoralists in southern Iran. *Pastoralism*, https://doi.org/10.1186/s13570-016-0056-y
248	Sulieman H.M., and Ahmed A.G.M. (2017). Mapping the pastoral migratory patterns under land appropriation in East Sudan: the case of the Lahaween Ethnic Group. *Geographical Journal*, https://doi.org/10.1111/geoj.12175
260	Chand R. (2017). Brokpa Yak Herders of Bhutan: A Study in Pastoral Livelihood Patterns, Transhumance and “Drukor”. *Perspectives on Geographical Marginality*, https://doi.org/10.1007/978-3-319-50998-3_12

269	Wangdi S., and Norbu N. (2018). Good fences are key to sustainable pasture management and harmonious pastoral society of Merak and Sakteng in Bhutan. *Pastoralism*, https://doi.org/10.1186/s13570-017-0106-0
271	Caplins L., and Halvorson S.J. (2017). Collecting Ophiocordyceps sinensis: an emerging livelihood strategy in the Garhwal, Indian Himalaya. *Journal of Mountain Science*, https://doi.org/10.1007/s11629-016-3892-8
272	Liao C., and Fei D. (2017). Pastoralist adaptation practices under non-governmental development interventions in Southern Ethiopia. *Rangeland Journal*, https://doi.org/10.1071/RJ16015
281	Ahearn A. (2018). Herders and hazards: covariate dzud risk and the cost of risk management strategies in a Mongolian subdistrict. *Natural Hazards*, https://doi.org/10.1007/s11069-017-3128-4
297	Karimi V., and Karami E., Keshavarz M. (2018). Vulnerability and Adaptation of Livestock Producers to Climate Variability and Change. *Rangeland Ecology and Management*, https://doi.org/10.1016/j.rama.2017.09.006
298	Buchanan A., and Reed M.G., Lidestav G. (2016). What’s counted as a reindeer herder? Gender and the adaptive capacity of Sami reindeer herding communities in Sweden. *Ambio*, https://doi.org/10.1007/s13280-016-0834-1
304	Klein J.A., and Hopping K.A., Yeh E.T., Nyima Y., Boone R.B., Galvin K.A. (2014). Unexpected climate impacts on the Tibetan Plateau: Local and scientific knowledge in findings of delayed summer. *Global Environmental Change*, https://doi.org/10.1016/j.gloenvcha.2014.03.007
305	Fernández-Giménez M.E. (2015). A shepherd has to invent: Poetic analysis of social-ecological change in the cultural landscape of the central Spanish Pyrenees. *Ecology and Society*, https://doi.org/10.5751/ES-08054-200429
309	Volpato G., and Howard P. (2014). The material and cultural recovery of camels and camel husbandry among Sahrawi refugees of Western Sahara. *Pastoralism*, https://doi.org/10.1186/s13570-014-0007-4
310	Wang J., and Wang Y., Li S., Qin D. (2016). Climate adaptation, institutional change, and sustainable livelihoods of herder communities in northern Tibet. *Ecology and Society*, https://doi.org/10.5751/ES-08170-210105

311	Dong S., and Yi S.L., Yan Z.L. (2016). Maintaining the Human-Natural Systems of Pastoralism in the Himalayas of South Asia and China. *Building Resilience of Human-Natural Systems of Pastoralism in the Developing World: Interdisciplinary Perspectives*, https://doi.org/10.1007/978-3-319-30732-9_3
315	He S., and Richards K. (2015). Impact of Meadow Degradation on Soil Water Status and Pasture Management-A Case Study in Tibet. *Land Degradation and Development*, https://doi.org/10.1002/ldr.2358
317	Misbah F., and Belay B., Haile A. (2015). Participatory definition of trait preference and pastorals' indigenous knowledge on goat breeding strategy around Aysaita District, Ethiopia. *Livestock Research for Rural Development*, https://doi.org/0
323	Mwangi M. (2016). Diverse drought spatiotemporal trends, diverse etic-emic perceptions and knowledge: Implications for adaptive capacity and resource management for indigenous Maasai-pastoralism in the rangelands of Kenya. *Climate*, https://doi.org/10.3390/cli4020022
326	Liao C., and Ruelle M.L., Kassam K.-A.S. (2016). Indigenous ecological knowledge as the basis for adaptive environmental management: Evidence from pastoralist communities in the Horn of Africa. *Journal of Environmental Management*, https://doi.org/10.1016/j.jenvman.2016.07.032
331	Sulieman H.M., and Siddig K.H.A. (2014). Climate change and rangeland degradation in Eastern Sudan: Which adaptation strategy works well?. *Nile River Basin: Ecohydrological Challenges, Climate Change and Hydropolitics*, https://doi.org/10.1007/978-3-319-02720-3_21
333	Tahmasebi A., and Ehlers E., Schetter C. (2013). Climate change and mountain pastoralism - The shahsevan of northwest Iran. *Erdkunde*, https://doi.org/10.3112/erdkunde.2013.04.02
340	Maiti S., and Jha S.K., Garai S., Nag A., Chakravarty R., Kadian K.S., Chandel B.S., Datta K.K., Upadhayay R.C. (2014). Adapting to climate change: Traditional coping mechanism followed by the Brokpa pastoral nomads of Arunachal Pradesh, India. *Indian Journal of Traditional Knowledge*, https://doi.org/0
341	Wu X., and Zhang X., Dong S., Cai H., Zhao T., Yang W., Jiang R., Shi Y., Shao J. (2015). Local perceptions of rangeland degradation and climate change in the pastoral society of Qinghai-Tibetan Plateau. *Rangeland Journal*, https://doi.org/10.1071/RJ14082

343	López-I-Gelats F., and Contreras Paco J.L., Huilcas Huayra R., Siguas Robles O.D., Quispe Peña E.C., Bartolomé Filella J. (2015). Adaptation strategies of Andean pastoralist households to both climate and non-climate changes. *Human Ecology*, https://doi.org/10.1007/s10745-015-9731-7
344	Bruegger R.A., and Jigjsuren O., Fernández-Giménez M.E. (2014). Herder Observations of Rangeland Change in Mongolia: Indicators, Causes, and Application to Community-Based Management. *Rangeland Ecology and Management*, https://doi.org/10.2111/REM-D-13-00124.1
356	Aryal S., and Maraseni T.N., Cockfield G. (2014). Sustainability of transhumance grazing systems under socio-economic threats in Langtang, Nepal. *Journal of Mountain Science*, https://doi.org/10.1007/s11629-013-2684-7
361	Takakura H. (2016). Limits of pastoral adaptation to permafrost regions caused by climate change among the Sakha people in the middle basin of Lena River. *Polar Science*, https://doi.org/10.1016/j.polar.2016.04.003
362	Molnár Z. (2014). Perception and Management of Spatio-Temporal Pasture Heterogeneity by Hungarian Herders. *Rangeland Ecology and Management*, https://doi.org/10.2111/REM-D-13-00082.1
363	Hopping K.A., and Yangzong C., Klein J.A. (2016). Local knowledge production, transmission, and the importance of village leaders in a network of Tibetan pastoralists coping with environmental change. *Ecology and Society*, https://doi.org/10.5751/ES-08009-210125
364	Turunen M.T., and Rasmus S., Bavay M., Ruosteenoja K., Heiskanen J. (2016). Coping with difficult weather and snow conditions: Reindeer herders' views on climate change impacts and coping strategies. *Climate Risk Management*, https://doi.org/10.1016/j.crm.2016.01.002
367	Singh N.J., and Bhatnagar Y.V., Lecomte N., Fox J.L., Yoccoz N.G. (2013). No longer tracking greenery in high altitudes: Pastoral practices of Rupshu nomads and their implications for biodiversity conservation. *Pastoralism*, https://doi.org/10.1186/2041-7136-3-16
370	Volpato G., and Lamin Saleh S., Nardo A. (2015). Ethnoveterinary of Sahrawi pastoralists of Western Sahara: camel diseases and remedies. *Journal of Ethnobiology and Ethnomedicine*, https://doi.org/10.1186/s13002-015-0040-4

372	Zampaligré N., and Dossa L.H., Schlecht E. (2014). Climate change and variability: perception and adaptation strategies of pastoralists and agro-pastoralists across different zones of Burkina Faso. *Regional Environmental Change*, https://doi.org/10.1007/s10113-013-0532-5
377	Russell J.M., and Ward D. (2016). HISTORICAL LAND-USE AND VEGETATION CHANGE IN NORTHERN KWAZULU-NATAL, SOUTH AFRICA. *Land Degradation and Development*, https://doi.org/10.1002/ldr.2476
379	Landau S.Y., and Muklada H., Abu-Rabia A., Kaadan S., Azaizeh H. (2014). Traditional Arab ethno-veterinary practices in small ruminant breeding in Israel. *Small Ruminant Research*, https://doi.org/10.1016/j.smallrumres.2014.01.004
382	Megersa B., and Markemann A., Angassa A., Ogutu J.O., Piepho H.-P., Valle Zárate A. (2014). Livestock Diversification: an Adaptive Strategy to Climate and Rangeland Ecosystem Changes in Southern Ethiopia. *Human Ecology*, https://doi.org/10.1007/s10745-014-9668-2
384	Wang J., and Brown D.G., Agrawal A. (2013). Climate adaptation, local institutions, and rural livelihoods: A comparative study of herder communities in Mongolia and Inner Mongolia, China. *Global Environmental Change*, https://doi.org/10.1016/j.gloenvcha.2013.08.014
389	Wu N., and Ismail M., Joshi S., Yi S.-L., Shrestha R.M., Jasra A.W. (2014). Livelihood diversification as an adaptation approach to change in the pastoral Hindu-Kush Himalayan region. *Journal of Mountain Science*, https://doi.org/10.1007/s11629-014-3038-9
395	Wario H.T., and Roba H.G., Kaufmann B. (2016). Responding to mobility constraints: Recent shifts in resource use practices and herding strategies in the Borana pastoral system, southern Ethiopia. *Journal of Arid Environments*, https://doi.org/10.1016/j.jaridenv.2015.12.005
398	Hewitt K. (2014). Glaciers in Human Life. *Advances in Asian Human-Environmental Research*, https://doi.org/10.1007/978-94-007-6311-1_13
400	Fernández-Giménez M.E., and Batkhishig B., Batbuyan B. (2012). Cross-boundary and cross-level dynamics increase vulnerability to severe winter disasters (dzud) in Mongolia. *Global Environmental Change*, https://doi.org/10.1016/j.gloenvcha.2012.07.001

406	Eira I.M.G., and Jaedicke C., Magga O.H., Maynard N.G., Vikhamar-Schuler D., Mathiesen S.D. (2013). Traditional Sámi snow terminology and physical snow classification-Two ways of knowing. *Cold Regions Science and Technology*, https://doi.org/10.1016/j.coldregions.2012.09.004
411	Shaoliang Y., and Ismail M., Zhaoli Y. (2012). Pastoral communities' perspectives on climate change and their adaptation strategies in the Hindukush-Karakoram-Himalaya. *Advances in Asian Human-Environmental Research*, https://doi.org/10.1007/978-94-007-3846-1_17
423	Spoon J. (2011). The Heterogeneity of Khumbu Sherpa Ecological Knowledge and Understanding in Sagarmatha (Mount Everest) National Park and Buffer Zone, Nepal. *Human Ecology*, https://doi.org/10.1007/s10745-011-9424-9
425	Fu Y., and Grumbine R.E., Wilkes A., Wang Y., Xu J.-C., Yang Y.-P. (2012). Climate Change Adaptation Among Tibetan Pastoralists: Challenges in Enhancing Local Adaptation Through Policy Support. *Environmental Management*, https://doi.org/10.1007/s00267-012-9918-2
426	Fernández-Giménez M.E., and Fillat F. (2012). Pyrenean pastoralists'observations of environmental change: An exploratory study in los Valles Occidentales of Aragón. *Pirineos*, https://doi.org/10.3989/
427	Fernández-Giménez M.E., and Estaque F.F. (2012). Pyrenean Pastoralists' Ecological Knowledge: Documentation and Application to Natural Resource Management and Adaptation. *Human Ecology*, https://doi.org/10.1007/s10745-012-9463-x
431	Joshi S., and Jasra W.A., Ismail M., Shrestha R.M., Yi S.L., Wu N. (2013). Herders' Perceptions of and Responses to Climate Change in Northern Pakistan. *Environmental Management*, https://doi.org/10.1007/s00267-013-0062-4
433	Sundstrom S., and Tynon J.F., Western D. (2012). Rangeland Privatization and the Maasai Experience: Social Capital and the Implications for Traditional Resource Management in Southern Kenya. *Society and Natural Resources*, https://doi.org/10.1080/08941920.2011.580420
437	Yan J., and Wu Y., Zhang Y. (2011). Adaptation strategies to pasture degradation: Gap between government and local nomads in the eastern Tibetan Plateau. *Journal of Geographical Sciences*, https://doi.org/10.1007/s11442-011-0904-z

446	Bulgakova T. (2010). Climate change, vulnerability and adaptation among Nenets reindeer herders. *Community Adaptation and Vulnerability in Arctic Regions*, https://doi.org/10.1007/978-90-481-9174-1_4
447	Zhang Q. (2012). THE DILEMMA OF CONSERVING RANGELAND BY MEANS OF DEVELOPMENT: EXPLORING ECOLOGICAL RESETTLEMENT IN A PASTORAL TOWNSHIP OF INNER MONGOLIA. *Nomadic Peoples*, https://doi.org/10.3167/np.2012.160108
453	Oteros-Rozas E., and Ontillera-Sánchez R., Sanosa P., Gómez-Baggethun E., Reyes-García V., González J.A. (2013). Traditional ecological knowledge among transhumant pastoralists in Mediterranean Spain. *Ecology and Society*, https://doi.org/10.5751/ES-05597-180333
458	Ghorbani M., and Azarniv H., Mehrabi A.A., Jafari M., Nayebi H., Seel K. (2013). The Role of Indigenous Ecological Knowledge in Managing Rangelands Sustainably in Northern Iran. *Ecology and Society*, https://doi.org/10.5751/ES-05414-180215
464	Zhang C., and Li W., Fan M. (2013). Adaptation of herders to droughts and privatization of rangeland-use rights in the arid Alxa Left Banner of Inner Mongolia. *Journal of Environmental Management*, https://doi.org/10.1016/j.jenvman.2013.04.053
467	Waudby H.P., and Petit S., Robinson G. (2013). Pastoralists' knowledge of plant palatability and grazing indicators in an arid region of South Australia. *Rangeland Journal*, https://doi.org/10.1071/RJ13021
471	Abate T., and Ebro A., Nigatu L. (2010). Traditional rangeland resource utilisation practices and pastoralists' perceptions on land degradation in south-east Ethiopia. *Tropical Grasslands*, https://doi.org/nan
484	Thevenin M. (2011). Kurdish Transhumance: Pastoral practices in South-east Turkey. *Pastoralism*, https://doi.org/10.1186/2041-7136-1-23
491	Haynes M.A., and Yang Y. (2013). Adapting to change: Transitions in traditional rangeland management of Tibetan yak herders in northwest Yunnan. *Environment, Development and Sustainability*, https://doi.org/10.1007/s10668-012-9426-9

493	Goldman M.J., and Riosmena F. (2013). Adaptive capacity in Tanzanian Maasailand: Changing strategies to cope with drought in fragmented landscapes. *Global Environmental Change*, https://doi.org/10.1016/j.gloenvcha.2013.02.010
494	Djoudi H., and Brockhaus M. (2011). Is adaptation to climate change gender neutral? Lessons from communities dependent on livestock and forests in northern Mali. *International Forestry Review*, https://doi.org/10.1505/146554811797406606
503	Reed M.S., and Dougill A.J., Taylor M.J. (2007). Integrating local and scientific knowledge for adaptation to land degradation: Kalahari rangeland management options. *Land Degradation and Development*, https://doi.org/10.1002/ldr.777
509	Adriansen H.K. (2002). Now sheep are our harvest: Dynamics in livelihood strategies of Fulani pastoralists in Senegal. *Geographica Hafniensia - Part A Phd Thesis*, https://doi.org/0
515	Verlinden A., and Kruger A.S. (2007). Changing grazing systems in central north Namibia. *Land Degradation and Development*, https://doi.org/10.1002/ldr.769
531	Homann S., and Rischkowsky B., Steinbach J., Kirk M., Mathias E. (2008). Towards endogenous livestock development: Borana pastoralists' responses to environmental and institutional changes. *Human Ecology*, https://doi.org/10.1007/s10745-008-9180-7
534	Homann S., and Rischkowsky B., Steinbach J. (2008). The effect of development interventions on the use of indigenous range management strategies in the Borana Lowlands in Ethiopia. *Land Degradation and Development*, https://doi.org/10.1002/ldr.845
541	Omotayo A.M., and Adu I.F., Aina A.B. (1999). The evolving sedentary lifestyle among nomadic pastoralists in the humid zone of Nigeria: implications for land-use policy. *International Journal of Sustainable Development and World Ecology*, https://doi.org/10.1080/13504509909470012
553	Huang J., and Bai Y., Jiang Y. (2009). Case Study 3: Xilingol Grassland, Inner Mongolia. *Rangeland Degradation and Recovery in China's Pastoral Lands*, https://doi.org/0
560	Xie Y., and Li W. (2008). WHY DO HERDERS INSIST ON OTOR? MAINTAINING MOBILITY IN INNER MONGOLIA. *Nomadic Peoples*, https://doi.org/10.3167/np.2008.120203

583	Bollig M., and Österle M. (2008). Changing Communal Land Tenure in an East African Pastoral System: Institutions and Socio-Economic Transformations among the Pokot of NW Kenya. *Zeitschrift fur Ethnologie*, https://doi.org/0
587	Davies J., and Bennett R. (2007). Livelihood adaptation to risk: Constraints and opportunities for pastoral development in Ethiopia's Afar Region. *Journal of Development Studies*, https://doi.org/10.1080/00220380701204422
591	Dong S.K., and Wen L., Zhu L., Lassoie J.P., Yan Z.L., Shrestha K.K., Pariya D., Sharma E. (2009). Indigenous yak and yak-cattle crossbreed management in high altitude areas of northern Nepal: A case study from Rasuwa district. *African Journal of Agricultural Research*, https://doi.org/0
608	Barrow E.G.C. (1991). Evaluating the effectiveness of participatory agroforestry extension programmes in a pastoral system, based on existing traditional values - A case study of the Turkana in Kenya. *Agroforestry Systems*, https://doi.org/10.1007/BF00141594
629	Roth E.A. (1996). Traditional pastoral strategies in a modern world: An example from northern Kenya. *Human Organization*, https://doi.org/10.17730/humo.55.2.14465222904843v4
647	Gentle, and P, Thwaites, R (2016). Transhumant Pastoralism in the Context of Socioeconomic and Climate Change in the Mountains of Nepal. *MOUNTAIN RESEARCH AND DEVELOPMENT*, https://doi.org/10.1659/MRD-JOURNAL-D-15-00011.1
648	Ulambayar, and T, Fernandez-Gimenez, ME, Baival, B, Batjav, B (2017). Social Outcomes of Community-based Rangeland Management in Mongolian Steppe Ecosystems. *CONSERVATION LETTERS*, https://doi.org/10.1111/conl.12267
651	Aryal, and S, Cockfield, G, Maraseni, TN (2016). Perceived changes in climatic variables and impacts on the transhumance system in the Himalayas. *CLIMATE AND DEVELOPMENT*, https://doi.org/10.1080/17565529.2015.1040718
656	Waudby, and HP, Petit, S, Robinson, G (2012). Pastoralists' perceptions of biodiversity and land management strategies in the arid Stony Plains region of South Australia: Implications for policy makers. *JOURNAL OF ENVIRONMENTAL MANAGEMENT*, https://doi.org/10.1016/j.jenvman.2012.07.012

657	Ouédraogo K., and Zaré A., Korbéogo G., Ouédraogo O., Linstädter A. (2021). Resilience strategies of West African pastoralists in response to scarce forage resources. *Pastoralism*, https://doi.org/10.1186/s13570-021-00210-8
660	Duenn, and P, Salpeteur, M, Reyes-Garcia, V (2017). RABARI SHEPHERDS AND THE MAD TREE: THE DYNAMICS OF LOCAL ECOLOGICAL KNOWLEDGE IN THE CONTEXT OF Prosopis juliflora INVASION IN GUJARAT, INDIA. *JOURNAL OF ETHNOBIOLOGY*, https://doi.org/nan
665	Marin, and A (2010). Riders under storms: Contributions of nomadic herders' observations to analysing climate change in Mongolia. *GLOBAL ENVIRONMENTAL CHANGE-HUMAN AND POLICY DIMENSIONS*, https://doi.org/10.1016/j.gloenvcha.2009.10.004
671	Siasiou, and A, Galanopoulos, K, Mitsopoulos, I, Ragkos, A, Laga, V (2018). Transhumant Sheep and Goat Farming Sector in Greece. *IRANIAN JOURNAL OF APPLIED ANIMAL SCIENCE*, https://doi.org/nan
673	Seijo, and F, Millington, JDA, Gray, R, Sanz, V, Lozano, J, Garcia-Serrano, F, Sanguesa-Barreda, G, Camarero, JJ (2015). Forgetting fire: Traditional fire knowledge in two chestnut forest ecosystems of the Iberian Peninsula and its implications for European fire management policy. *LAND USE POLICY*, https://doi.org/10.1016/j.landusepol.2015.03.006
679	Oba, and G, Byakagaba, P, Angassa, A (2008). PARTICIPATORY MONITORING OF BIODIVERSITY IN EAST AFRICAN GRAZING LANDS. *LAND DEGRADATION & DEVELOPMENT*, https://doi.org/10.1002/ldr.867
680	Oteros-Rozas, and E, Martin-Lopez, B, Gonzalez, JA, Plieninger, T, Lopez, CA, Montes, C (2014). Socio-cultural valuation of ecosystem services in a transhumance social-ecological network. *REGIONAL ENVIRONMENTAL CHANGE*, https://doi.org/10.1007/s10113-013-0571-y
681	Fernandez-Gimenez, and ME, Batkhishig, B, Batbuyan, B, Ulambayar, T (2015). Lessons from the Dzud: Community-Based Rangeland Management Increases the Adaptive Capacity of Mongolian Herders to Winter Disasters. *WORLD DEVELOPMENT*, https://doi.org/10.1016/j.worlddev.2014.11.015

684	Tumenjargal, and S, Fassnacht, SR, Venable, NBH, Kingston, AP, Fernandez-Gimenez, ME, Batbuyan, B, Laituri, MJ, Kappas, M, Adyabadam, G (2020). Variability and change of climate extremes from indigenous herder knowledge and at meteorological stations across central Mongolia. *FRONTIERS OF EARTH SCIENCE*, https://doi.org/10.1007/s11707-019-0812-6
721	Marchina, and C (2021). Nomad's land, no man's land?. *NOMADIC PASTORALISM AMONG THE MONGOL HERDERS: Multispecies and Spatial Ethnography in Mongolia and Transbaikalia*, https://doi.org/10.5117/9789463721424_CH01
728	Fassnacht, and SR, Allegretti, AM, Venable, NBH, Fernandez-Gimenez, ME, Tumenjargal, S, Kappas, M, Laituri, MJ, Batbuyan, B, Pfohl, AKD (2018). Merging Indigenous Knowledge Systems and Station Observations to Estimate the Uncertainty of Precipitation Change in Central Mongolia. *HYDROLOGY*, https://doi.org/10.3390/hydrology5030046
730	Mijiddorj, and TN, Ahearn, A, Mishra, C, Boldgiv, B (2019). Gobi Herders' Decision-Making and Risk Management under Changing Climate. *HUMAN ECOLOGY*, https://doi.org/10.1007/s10745-019-00112-9

Supplementary Information B

Table S2. Definition of variables used in the systematic literature review

Variable		Definition	Format
**Metadata**			
Study area	Location	Indicate name(-s) of regions where research took place	Coordinates
	Country(-ies)	Indicate country(-ies) where research took place	Name of the country(-ies)
	GPS	The Global Positioning System coordinates that precisely define the location of the study area	Latitude and Longitude
	Climate zones	Climate types as defined by Koeppen-Geiger climate classification (if not mentioned, it is extrapolated from location)	1=Dry<br>2=Continental<br>3=Temperate<br>4=Tropical<br>5=Polar
Study group	Ethnic groups	Indicate which ethnic groups were respondents in this study	The name of the ethnic group. if not specified, write NA
	Indigenous Peoples	Whether the authors conceptualize the respondents as indigenous peoples	0=No<br>1= Yes
	Type of pastoralism	Nomadism: characterized by the regular movement of herds to new pastures.<br>Transhumance: involving seasonal migration between fixed pastures.<br>Agro-pastoralisim: combines crop farming with pastoralism.<br>Sedentarism: indicating settled herding with limited livestock movement.	1= Nomadism<br>2=Transhumance<br>3= Agro-pastoralisim<br>4= Sedentarism
	Type of herd (can be multiple)	The type of herd the local communities manage.	1= Cattle<br>2= Sheep<br>3= Goat<br>4= Camel<br>5= Horse<br>6= Reindeer<br>7=Yak<br>8=Other
Methods	Research Methods	Method of data collection	1= Community Mapping<br>2= Focus Group Discussions<br>3= Free list<br>4= Interviews<br>5= Participant Observation<br>6= Surveys<br>7=other
**Pastoral Traditional Knowledge Domains**			

PTK domains	Livestock-related knowledge	This knowledge includes understanding the nutritional needs, well-being, grazing preferences, and unique characteristics of the livestock.	0=No<br>1= Yes
	Forage and plant-related knowledge	This knowledge involves the identification of plants, a detailed understanding of their characteristics, and their utilization.	0=No<br>1= Yes
	Landscape-related knowledge	This knowledge is about the observation and understanding of the specific landscapes where pastoralists live.	0=No<br>1= Yes
	Climate and weather-related knowledge	This knowledge refers to information about the local climate, weather patterns, and seasonal variations that impact pastoral activities.	0=No<br>1= Yes
	Social-cultural knowledge	This knowledge is about the cultural traditions, social institutions, and community dynamics specific to pastoral societies.	0=No<br>1= Yes
	Herd mobility	This knowledge refers to the movement of livestock groups from one location to another for different purposes.	0=No<br>1= Yes
	Herd breeding	This knowledge is specific to the selective breeding and reproduction of livestock within a specific pastoral context.	0=No<br>1= Yes
	Herd diversification	This knowledge focuses on the trade-offs of diversifying the composition of the livestock.	0=No<br>1= Yes
	Landscape management	This knowledge includes the traditional insights and techniques developed for interacting with their environment.	0=No<br>1= Yes
	Other	This domain includes knowledge that does not fit in any of the domains, such as holistic knowledge.	0=No<br>1= Yes
**Pastoral Traditional Knowledge Functions**			
PTK functions	Main functions (can be multiple)	Ecological function includes various aspects such as managing ecosystem health, preventing<br>weather and climate variations and contributing to ecosystem recovery.<br><br>Economic function includes functions related to enhancing the efficiency and sustainability of pastoralists' livelihoods.<br><br>Social-cultural function includes functions contribute to the maintenance of pastoral communities' cultural integrity and social structures.	1=Ecological function<br><br>2=Economic function<br><br>3=Social-cultural function

	Subfunctions (can be multiple)	Biodiversity conservation and ecosystem monitoring emphasizes the role of knowledge in monitoring and maintaining ecosystem health, and in conserving local species, and the genetic diversity of livestock breeds.<br><br>Sustainable resource management emphasizes the role of knowledge in maintaining sustainable use of the natural resources and ensuring long-term sustainability of rangelands.<br><br>Climate adaptation and resilience emphasizes the role of knowledge in observing and predicting weather patterns and preparing for the varying climatic conditions and environmental stressors.<br><br>Ecosystem restoration and regeneration emphasizes the role of knowledge in contributing to the recovery and regeneration of ecosystems.<br><br>Livelihood support and resource optimization emphasizes the role of knowledge in optimizing the use of limited resources, including water and forage, to support livelihoods.<br><br>Risk management and disaster reduction emphasizes the role of knowledge in mitigating the impacts of natural disasters and environmental fluctuations.<br><br>Enhancing livestock productivity emphasizes the role of knowledge in improving livestock productivity.<br><br>Cultural identity and heritage emphasizes the role of knowledge in preserving the cultural heritage and traditional practices that define community identity.<br><br>Social cohesion and community governance emphasizes the role of knowledge in strengthening community bonds through shared decision-making,	1.1=Biodiversity conservation and ecosystem monitoring<br><br>1.2=Sustainable resource management<br><br>1.3=Climate adaptation and resilience<br><br>1.4=Ecosystem restoration and regeneration<br><br>2.1=Livelihood support and resource optimization<br><br>2.2=Risk management and disaster reduction<br><br>2.3=Enhancing livestock productivity<br><br>3.1=Cultural identity and heritage<br><br>3.2=Social cohesion and community governance<br><br>3.3=Conflict resolution and cooperation

		resource-sharing, and collective management of resources.<br><br>Conflict resolution and cooperation emphasizes the role of knowledge in resolving disputes over resources and fostering cooperation in herding practices.	
